# Liquid Metal-Based Flexible Bioelectrodes for Management of In-Stent-Restenosis: Potential Application

**DOI:** 10.3390/bios13080795

**Published:** 2023-08-07

**Authors:** Xilong Zhang, Lei Li, Zhongshan Deng

**Affiliations:** 1 Key Laboratory of Cryogenics, Technical Institute of Physics and Chemistry, Chinese Academy of Sciences, Beijing 100190, China; zhangxilong21@mails.ucas.ac.cn; 2 School of Future Technology, University of Chinese Academy of Sciences, Beijing 100049, China; 3 Plastic Surgery Hospital, Chinese Academy of Medical Sciences, Beijing 100144, China

**Keywords:** vascular stent, liquid metal, in-stent restenosis, irreversible electroporation, biosensor

## Abstract

Although vascular stents have been widely used in clinical practice, there is still a risk of in-stent restenosis after their implantation. Combining conventional vascular stents with liquid metal-based electrodes with impedance detection, irreversible electroporation, and blood pressure detection provides a new direction to completely solve the restenosis problem. Compared with conventional rigid electrodes, liquid metal-based electrodes combine high conductivity and stretchability, and are more compliant with the implantation process of vascular stents and remain in the vasculature for a long period of time. This perspective reviews the types and development of conventional vascular stents and proposes a novel stent that integrates liquid metal-based electrodes on conventional vascular stents. This vascular stent has three major functions of prediction, detection and treatment, and is expected to be a new generation of cardiovascular implant with intelligent sensing and real-time monitoring.

## 1. Introduction

Cardiovascular disease (CVD) is the most deadly disease in the world, accounting for approximately one-third of global deaths [1]. Angiemphraxis is one of the causes of CVD. The implantation of vascular stents can effectively unblock blood vessels. However, the endothelial damage caused by balloon dilation induces a healing response that leads to in-stent restenosis (ISR, which is defined as a restenosis of >50% of the lumen diameter of the entire in-stent or 5 mm segment at both ends of the stent after stent implantation as detected by coronary angiography).

Vascular wall injury after percutaneous transluminal coronary intervention (PCI) causes an associated inflammatory response that contributes to fibroblast and smooth muscle cell proliferation, which is the main mechanism for the occurrence of in-stent restenosis [2]. To reduce neointimal hyperplasia, the first generation of drug-eluting stents (DESs) was introduced. However, in the following four years, there was no difference in mortality or myocardial infarction rates between DES and bare-metal stents (BMSs) [3]. Therefore, after extensive clinical data analysis, the investigators proposed the second generation of DESs, which improved on the first generation in three main ways, to make the restenosis rate less than 10% [4]. However, because the bare stent remains as foreign matter for a long time, it still causes late thrombosis and very late adverse events, with a lethality rate of 45% [5]. As a result, researchers embarked on the exploration and study of biodegradable stents (BDSs). Experiments have shown that BDSs can restore the contractile function and increase the lumen diameter of the stented segment of the vessel, thus further reducing the angina burden [6]. Currently, BDSs are mainly classified into polymeric and metallic types. However, metal-based BDSs have problems in the choice of materials, such as mechanical strength and flexibility game [7], while polymer-based BDSs have problems such as radioactivity, reduction of radial force, and lack of lateral plasticity [8]. In recent years, Bussooa et al. [9] proposed a microstructural biosensor that can detect impedance and induce electrically mediated apoptosis of vascular smooth muscle cells (SMCs). This design inhibits SMC proliferation while allowing real-time remote monitoring of intravascular cell growth for both diagnostic and therapeutic purposes. The combination of irreversible electroporation (IRE) technology and vascular stents provides a new idea to reduce the rate of ISR. It has been shown that electrical stimulation not only induces apoptosis [10], but also induces a higher proportion of apoptotic cell death by electrical stimulation compared to drug-induced SMC death [8]. Since apoptosis is more conducive to maintaining the stability of the internal environment than cell necrosis, it is highly feasible to address the problem of ISR by applying pulsed voltage to inhibit the excessive proliferation of smooth muscle cells. However, in the current study, the electrodes used for electrically induced apoptosis are mostly rigid electrodes [9]. Rigid electrodes as long-term implants have the possibility of fracture, due to human twisting or external impact, so the choice of electrode material is crucial.

Liquid metals (LMs) are materials with low melting points, such as gallium-based alloys and bismuth-based alloys, and include the resulting composites [11]. The LMs mentioned below are all gallium-based eutectic alloys, including EGa_68.5_In_31.5_ (melting point 15.7 °C) and EGa_68.5_In_21.5_Sn_10.0_ (melting point 12 °C). Flexible electrodes prepared from LM have good compliance to alleviate mechanical mismatch at the tissue–electrode interface, which plays a key role in long-term implantation stability. In 2018, R. David et al. [12] developed an LM electrode microarray that allows direct electrical stimulation of biological tissues, and in vitro experiments were performed to record the frequency behavior of electrode coupling over the 1 Hz–1 MHz frequency band with input amplitude between 250 mV and 2 V. The first effort to introduce gallium-based LM components into the field of implantable electrical stimulation systems was demonstrated. The following year, Li et al. [13] prepared gallium-indium-tin LM-based electrodes encapsulated in a soft PDMS, and formed IDE capacitors. The IDE capacitor was then integrated with a capacitive sensing chip, which could capture the respiratory rate due to human chest motion, using the proximity sensing effect. These studies paved the way for the application of LM-based bioelectrodes for continuous health monitoring. However, all the above are in vitro experiments. Jiang et al. [14] proposed a strategy to prepare highly stretchable neural electrode arrays based on LM conductors screen printed onto PDMS substrates, and demonstrated the great potential of liquid metal bioelectrodes for electrical signal transmission in a series of in vivo evaluation experiments. Therefore, LM-based flexible electrodes can be integrated into a vascular stent to form an intelligent implantable medical device. The development of the DES and BDS will also promote the development of this new type of vascular stent.

This perspective will focus on how ISR can be addressed by a novel vascular stent that integrates LM-based flexible electrodes. The functions of LM-based electrodes include impedance detection, blood pressure monitoring, and electrical stimulation. These functions allow the novel vascular stent to be effective for prediction, detection, and treatment. It greatly contributes to the development of a new generation of intelligent vascular stents.

## 2. Techniques for the Treatment of In-Stent Restenosis

The previous study concluded that the process of ISR formation after PCI is divided into five stages: acute or subacute plaque shedding, vessel wall elasticity retraction, vascular remodeling, de novo intimal hyperplasia and in-stent atherosclerosis [15]. Among these, neointimal hyperplasia is the culprit regarding ISR, so the choice of drugs in DES is currently based on the inhibition of endothelial cell proliferation.

### 2.1. Drug-Eluting Stent

**Figure 1:**
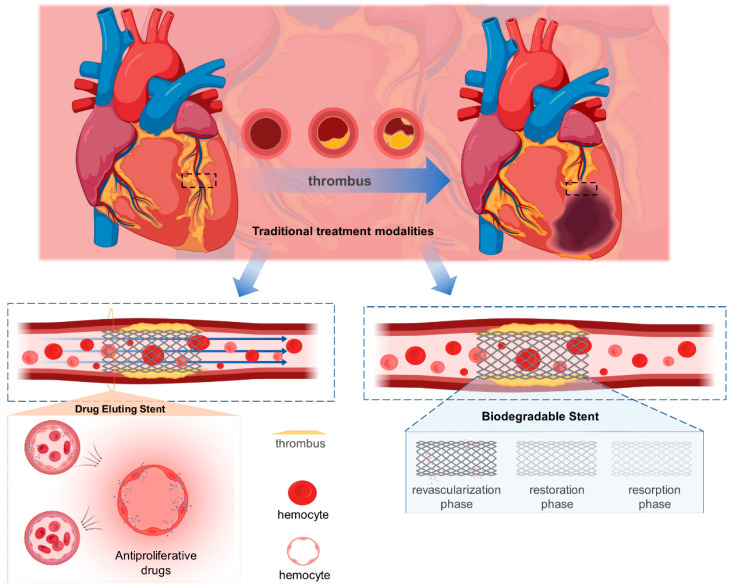
Schematic diagram of the principle of drug-eluting stents and degradable stents.

The DES reduces the risk of ISR by loading the BMS surface with drugs that can inhibit endothelial proliferation, and its principle is shown in Figure 1. A wide variety of DESs are currently undergoing animal experiments and preclinical trials, but the only DESs on the market are sirolimus (rapamycin)-eluting stents and paclitaxel(PTX)-eluting stents, whose inhibition mechanism is that PTX inhibits dormancy-resistant microtubules, thereby inhibiting cell division, while sirolimus inhibits the transition of cell style from G1 phase to S phase [16,17,18,19].

#### 2.1.1. First Generation

The first generation of DBSs is represented by the sirolimus-coated Cypher stent and the paclitaxel-coated Taxus stent, both with stainless steel as the backbone, relatively thick stent walls, and non-degradable polymeric coating materials. (Table 1 summarizes the company names of all commercial stents that appear in this perspective). After a series of data analyses, it was found that the lethality rate or the incidence of myocardial infarction was almost the same between drug-eluting stents and bare-metal stents, within four years. In addition, Gregg W. Stone et al. [20] analyzed four double-blind trials of these two types of stents in 2007, and their results are shown in Table 2; they found that the use of drugs, although effective in avoiding restenosis, increased the probability of late thrombosis. In this regard, the investigators continued to explore in depth the three aspects of scaffold, drug and polymer coating.

#### 2.1.2. Second Generation

In 2008, three second-generation stents were first approved, the Taxus Liberte stent, the Medtronic Endeavor and the Abbott XIENCE V. The Taxus Liberte stent changed the geometry of the stainless steel from open-hole to closed-hole, based on the Taxus Express stent, but clinical trials showed that this improvement did not have significant effect. The clinical trials showed no significant advantage to this improvement [21]. Instead of stainless steel, a cobalt-chromium alloy with excellent mechanical properties has been chosen for the stent skeleton of Endeavor and XIENCE V. In addition, the Medtronic Endeavor stent was designed to incorporate the sirolimus analogue zotarolimus, as well as a phosphorylcholine (PC) polymer bionic coating. And the Abbott XIENCE V stent combines another sirolimus analogue, everolimus, and a polymeric coating consisting of PVDF-HFP. Both were then subjected to randomized, single-blind clinical studies, the results of which demonstrated a significantly lower incidence of late-acquired incomplete stent apposition with the zotarolimus-eluting stent [22]. There was no significant change in the incidence of thrombosis with the XIENCE V stent, but the rate of target lesion revascularization and major adverse cardiovascular events decreased significantly [23].

Compared to the first generation of DESs, the second generation of DESs not only use sirolimus derivatives that are less cytotoxic and reduce local inflammatory effects, but a polymer with better bio-solubility is also selected, thus reducing local inflammatory reactions or allergic reactions. In addition, the skeleton material of the stent has been changed, and a stronger alloy material chosen to make the stent wall thickness thinner [24].

#### 2.1.3. Optimization of DES

**Figure 2:**
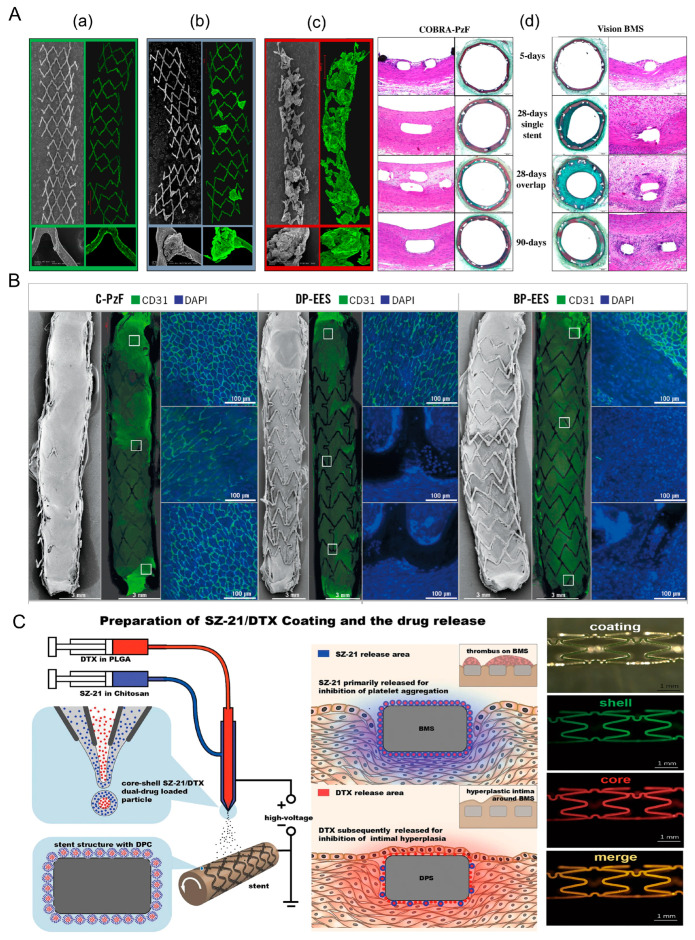
(**A**) Ex-vivo arteriovenous porcine shunt model. Representative images derived from confocal microscopy (10× and 20× magnifications) and scanning electron microscopy (15× and 200× magnifications) in a porcine ex vivo AV-shunt model. Panel (**a**) shows the COBRA PzF nano-coated stent. Panel (**b**) shows the COBRA bare metal stent. Panel (**c**) shows the Multilink Vision bare metal stent. (**d**) Representative light microscopy images of a porcine ex vivo arteriovenous shunt model and implantation of cobra- pzf and Vision BMS stents in normal porcine coronary arteries for 5, 28 and 90 days [26]. (**B**) Endothelial coverage of different stents [27]. (**C**) Schematic diagrams of material design and function hypothesis as well as evaluation of the surfaces of different stent coatings [28].

Although DES is currently the most widely used vascular stent in clinical practice, practical results show that DES can still be optimized toward reducing the thickness of the stent, enhancing the drug fit, and improving the structure of the stent surface. Hiroyoshi et al. [25] selected a special inorganic high-molecular-weight polymer Polyzene-F (PzF), which is a nano-thin bionic surface coating, as the coating material. As shown in Figure 2A, since PzF is a fluoropolymer, it can inhibit the formation of thrombus while having anti-inflammatory properties. In addition, clinical studies have shown (as shown in Figure 2B) that the restenosis rate is significantly lower with PzF compared to bare metal stents. Therefore, the PzF coating can act as a barrier between the device, the endothelial surface, and the circulating elements in the blood. This suggests that drug-free nano-coatings can be a new direction to think about, especially for patients with low platelet levels, as platelets have difficulty adhering to PzF nanocoatings [26]. In addition, this coating has the potential to be loaded with drugs [27].

Since the currently used drugs are unable to efficiently inhibit both platelet aggregation and endocardial proliferation, researchers have proposed a new class of drug coatings that can load and control the release of multiple drugs. Du et al. [28] designed a nanocoating with a crustal structure. As shown in Figure 2C, the surface is platelet glycoprotein IIb/IIIa receptor monoclonal antibody SZ-21 and the core is the antiproliferative drug doxorubicin (DTX). Li et al. [29] used coatings containing different drugs, and these modified stents have been more successful in in vitro experiments.

In addition, the drug type, stent skeleton material and stent structure design can be optimized in the future. Thus, thrombosis and ISR can be inhibited while achieving vascular reendothelialization. These optimization directions may also facilitate the development of vascular scaffolds with integrated flexible LM bioelectrodes.

### 2.2. Biodegradable Stent

Both the BMS and the DES inevitably leave a “permanent” metallic skeleton in the vessel, which affects the compliance of the diseased vessel segment. This is why the BDS has become a focus of interest for researchers. There are three phases after stent implantation: the support phase, the degradation phase and the recovery phase. The support phase allows for drug release to help the stent better adapt to the vessel. During the support phase, it is critical that metallic and polymeric BDSs maintain mechanical stability and biocompatibility.

#### 2.2.1. Metal Degradable Stent

Metal-based BDS candidates are dominated by Mg, Fe, and Zn, benefiting from their simultaneously excellent mechanical properties and adequate biocompatibility. However, they also have dynamic interaction surfaces and continuous degradation, releasing various by-products that continuously change the device/tissue interface, which makes metal-based stents challenging to evaluate [30].

Successful data were obtained in actual clinical trials. The data showed that the Mg-based scaffold had the weakest inflammatory response after implantation of all three types of BDS, but it was the least intense [7]. In contrast, the Fe-based inflammatory response was the strongest, due to the greater toxicity of hydroxyl radicals generated by Fe during the corrosion process [31,32]. However, iron nitride scaffolds can effectively improve the intensity of the inflammatory response [33]. Compared to Mg and Zn, Fe has the most excellent mechanical properties, and therefore the iron-based stent is the first metal BDS to be fabricated and evaluated in vivo [34]. However, too high a mechanical strength can make the degradation of Fe too slow, although the addition of Mn elements can accelerate the degradation rate of Fe-based scaffolds. However, it will make more soluble toxic corrosion products in the scaffold [35]. Zn, on the other hand, combines the properties of Mg and Fe, which have a more favorable in vivo corrosion rate as well as appropriate biocompatibility [36,37]. However, Zn is an HCP (hexagonal close-packed) structure that is difficult to deform and cannot be effectively used as a scaffold for atherosclerotic lesions, and thus researchers have developed Zn-based materials with higher mechanical strength by alloying them [38,39].

#### 2.2.2. Polymer-Based Degradable Stent

The first biodegradable stent was the Igaki-Tamai (drug-free coated stent made of polymerized lactic acid) developed in Japan, and its clinical results have led a large number of investigators to join the BDS study [40]. The first BDS on the market was Abbott’s Absorb, a stent made from a combination of polymerized lactic acid and everolimus coating. However, the AIDA study published in NEJM in 2017 showed a much greater risk of thrombosis in the Absorb group (3.5%) than in the CoCr-EES group (0.9%) [41]. Therefore, after Absorb was withdrawn from the market in 2017 to reduce the thrombotic risk by optimizing the implantation technique, Absorb IV required the placement of BDS on the basis of adequate pre-expansion as well as post-expansion, and its clinical results showed that the thrombotic risk was successfully reduced to 0.6% [42].

The biggest drawback of polymers compared to metals is their weak mechanical strength. The thickness of the stent beam is limited by the size of the vessel, so the choice of stent material is crucial, and Fantom is made of polytyrosine-derived polycarbonate (PTD-PC). Compared with Absorb, Fantom is lighter and thinner. The stent has better elasticity and radial support properties, and the Fantom series is currently in the clinical research phase [43].

#### 2.2.3. Optimization of BDS

**Figure 3:**
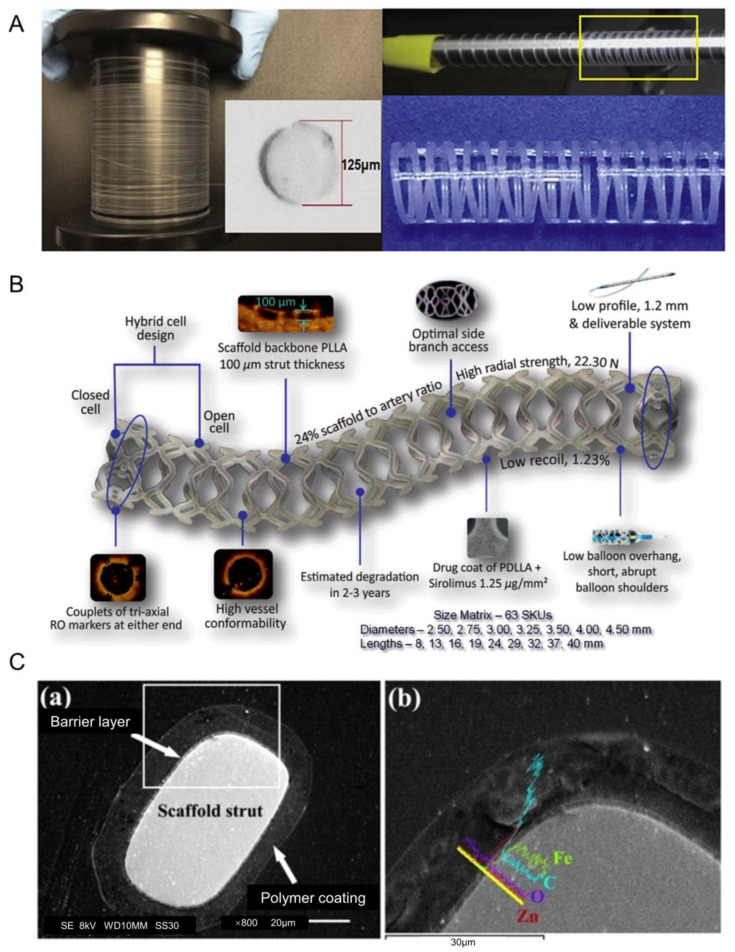
(**A**) Schematic diagram of the structure of the Mirage bioresorbable microfiber sirolimus (BRMS) eluting stent [44]. (**B**) Design of the MeRes100 bioresorbable vascular scaffold stent platform [45]. (**C**) The strut cross-section (**a**) structure and (**b**) composition of the IBS scaffold. The area of (**b**) is an enlargement of the area in the white rectangle of (**a**) [51].

The BDS embodies the new concept of “intervention without implantation”. For polymeric BDSs, researchers have made many attempts to reduce the thickness of the stent without affecting its drug delivery performance. As shown in Figure 3A, Mirage’s stent structure is designed in a spiral shape, which can provide better flexibility [44]. As shown in Figure 3B, the MeRes100 uses a new holder structure and limits the thickness of the holder beam to 100 μm to achieve better radial support [45]. AMSorb is made of the same polymerized lactic acid, but uses a unique 3D printing technique to create the stent, resulting in a more rounded cross-section of the stent beam and a reduced cross-sectional area of the stent (approximately 50% less than Absorb), which may reduce some risk of thrombosis [46]. In addition, Liu et al. [47] found that the use of shape-memory polymers as stent beams can reduce the invasiveness of the stent and provide more stable swelling performance by performing simulation analysis. Therefore, in the future, polymer-based BDSs can be optimized in terms of surface and structural design, material selection and distribution of stents.

Mg and its alloys are considered promising alternatives to permanent biomedical materials, due to their tunable mechanical strength and appropriate biodegradability, which can be further tuned by surface coating techniques [48]. The in vivo safety of Fe scaffolds (>99.8% Fe) was initially demonstrated by implantation into rabbit aorta in 2001 [34], but Fe-based stents require a higher degradation rate, while both Zn and Mg have faster degradation rates than Fe [49]. Although the degradation rate and biocompatibility of Zn is excellent, the mechanical strength of Zn is too low [50]. Therefore, BDS should consider combining multiple materials in the future and not be limited to one type of metal and its alloys. For example, the IBS stent, which has recently entered the clinical phase, has a structure as shown in Figure 3C. The material chosen is iron nitride manufactured by plasma-nitrogen laser cutting of pure iron, which is then plated with Zn and finally coated with rapamycin at 235 μg/cm^2^. The entire stent beam is only 53 μm thick, and clinical trials of the IBS are expected to end in 2024 [51]. Therefore, metal-based BDSs can be surface treated and metal alloyed, with improved manufacturing processes in the future. And the optimized BDS can be used as the backbone of vascular scaffolds, with integrated flexible LM bioelectrodes.

## 3. The Potential of Liquid-Metal-Based Electrodes Combined with Vascular Stents

There are many different types of coronary stents for clinical applications, each with its own characteristics. The BMS is now obsolete, due to the high rate of in-stent restenosis. Most of the DESs have been used for clinical treatment, while most of the BDSs are in clinical trials. However, whether DES or BDS, “silent” late in-stent occlusions and thrombosis still exist. Therefore, future technologies should focus on how to solve this problem. Vascular stents should not only have a therapeutic function, but also a diagnostic and predictive function.

### 3.1. Advantages of Liquid-Metal-Based Electrodes

LM-based flexible electrodes are more suitable as long-term implants than rigid electrodes. Bussooa et al. [9] proposed a microstructured biosensor for the diagnosis and treatment of vascular blockage with electrodes made of titanium (Ti) and gold (Au). Experimental results showed that the electrodes could effectively induce apoptosis in smooth muscle cells, and thus control the thickness of neointimal proliferation. However, the biosensor has not been applied to vascular scaffolds, and is present as a graft. Hoare et al. [52] improved the design of Bussooa et al. They moved the sensor from a fracture-prone glass substrate to a silicon substrate with an electrode material of choice Pt and Au. The silicon biosensor was integrated into a custom Nitinol stent that was designed to keep the sensor in place during radial expansion without deformation. However, as a long-term implant, there is a risk of misalignment and fracture of rigid electrodes when the body is subjected to external forces. The introduction of flexible electrodes becomes particularly important. LM-based flexible electrodes not only have no LM leakage behavior after thousands of torsions and bends, but all circuits still maintain excellent electrical conductivity [53].

**Figure 4:**
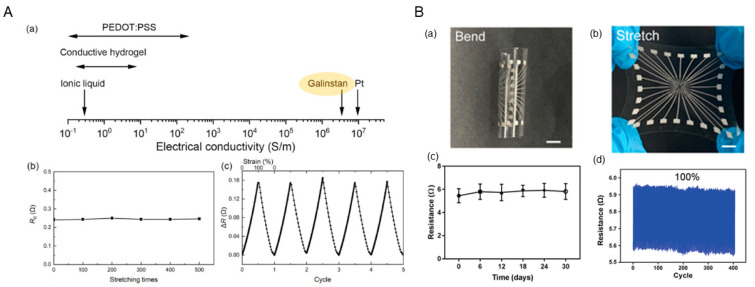
(**A**) The resistance and electrochemical properties: (**a**) the electrical conductivity of Galinstan, Pt, and commonly used flexible conductors; (**b**) the resistance of the LM electrode after various numbers of stretch–relax cycles; (**c**) the resistance changes of the LM electrode during cyclic stretching [54]. (**B**) Snapshots of the SEA with high flexibility (**a**) and stretchability (**b**); (**c**) Resistance of the conductive with PBS incubation over a course of 30 days; (**d**) Impedance of the Pt-coated electrodes and LM electrodes with the same area as a control [55].

LM-based electrodes with stretchability are more adaptable to the implantation process of vascular stents. Vascular stents need to be delivered into the vessel at the site of the lesion along the guidewire during implantation. The wall of the vessel is pushed outward by balloon inflation to restore vessel patency. When the balloon is inflated, the vascular stent is also stretched. This requires that the bioelectrodes integrated into the vascular stent have a certain degree of stretch. As shown in Figure 4A, LM-based bioelectrodes can combine high conductivity and stretchability compared to bioelectrodes made of conductive materials such as conductive polymers, carbon nanotubes, hydrogels, and solid metals. Tang et al. [54] developed a cuff electrode using a gallium-based LM conductor. The experimental results showed that the flexibility and tensile properties of the electrode can match those of biological tissues. Dong et al. [55] prepared a highly stretchable electrode array (SEA) based on an LM-polymer conductor. As shown in Figure 4B, the electrode not only exhibited high stretchability (≈100%) but also showed excellent cycling stability (>400 cycles). Therefore, LM-based electrodes can be implanted with the vascular stent and remain in the vessel for a long period of time.

LM electrodes based on microfluidic technology can be used for blood pressure monitoring, to determine if there is a vessel blockage near the implanted segment. Zhou et al. [56] designed an LM-based capacitive soft pressure microsensor for measuring pressure in microchannels. The sensor was also implanted into the carotid artery of rabbits for vascular measurements. Experiments showed that the capacitive pressure sensor was sensitive enough to detect pressure fluctuations in animals such as rabbits, and can therefore be used as an implantable device for blood pressure monitors.

### 3.2. Challenges of Liquid-Metal-Based Electrodes for Vascular Stent

LM-based bioelectrodes also have many challenges in the development process. LM exhibits degradation behavior in acidic environments [57]. Encapsulation of the LM is required to ensure the long-term stability of the bioelectrode during implantation. Due to the small encapsulation area, the encapsulation materials can be conductive materials such as conductive polymers, hydrogels and silver nanosheets. In addition, although there are many ways to prepare LM-based electrodes, the final thickness of LM-based electrodes made by microfluidic technology or screen printing technology is hardly less than 100 μm. In order to broaden its application occasions, the future research should be developed in the direction of ultra-thin LM-based electrodes. Currently, LM micro-networks with a thickness of 2 μm have been fabricated by researchers using the thermal deposition method [58]. However, long-term in vivo implantation experiments on such electrodes are lacking. Therefore, the long-term implantability and stretchability of LM-based electrodes produced by thermal deposition should be explored in the future. LM-based electrodes prepared by microfluidics will be preferred when the thickness permits. This is due to the fact that the electrodes prepared by this technique are easy to encapsulate and have a higher conductivity than electrodes prepared by the screen printing method.

## 4. Perspective

### 4.1. Structural Design of Integrated Vascular Stent Based on Liquid Metal

**Figure 5:**
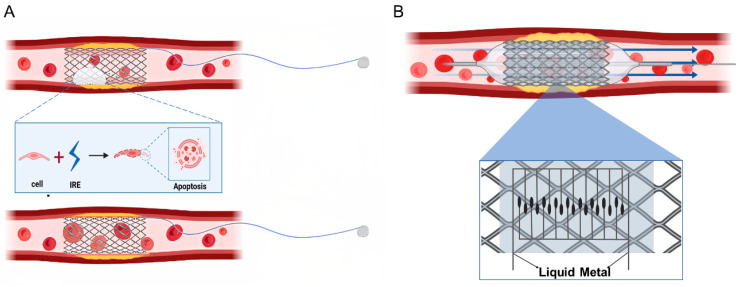
(**A**) Schematic diagram of the principle of irreversible perforation. (**B**) Schematic diagram of the structure of a vascular scaffold based on LM-based bioelectrodes.

Gallium-based LMs are often used as bioelectrode materials due to their excellent properties. Foremny et al. [59] first measured the biocompatibility of LM as a flexible implant material and later also experimentally verified that LMs can be successfully integrated into scalable devices. Zhu et al. [60] used a gallium–indium alloy LM as a microelectrode and integrated it into a microfluidic chip to capture and stretch red blood cells. This work not only demonstrated the potential of LMs for microfluidic applications, but also paved the way for using LMs as microelectrodes for cell manipulation. Cheng et al. [61] designed an electronic blood vessel made of biodegradable polymer and LM. The vessel not only had excellent biocompatibility in the vascular system, but also showed excellent patency 3 months after implantation in a rabbit model. In this experiment, LM-based electrodes have been successfully used for cell electrotransfection (reversible electroporation). Irreversible electroporation is expected to be achieved by adjusting the magnitude of the voltage, thus inducing apoptosis in smooth muscle cells (as shown in Figure 5A.)

Therefore, it is feasible to integrate a flexible LM-based bioelectrode into a vascular stent. The structure of the new vascular stent is schematically shown in Figure 5B, where the outer surface of the balloon is wrapped by an integrated vascular stent. The skeleton of the vascular scaffold can be made of nickel–titanium alloy, cobalt–chromium alloy, stainless steel and biodegradable materials. The surface of the skeleton can be modified or coated with a drug to inhibit the incidence of ISR. A balloon is delivered to drive the integrated vascular stent percutaneously into the vasculature and to the site of the lesion to unblock the vessel. If the skeleton material is non-biodegradable, the biosensor is located between the skeletal structure and the balloon, allowing for health monitoring after deployment. If the skeleton material is biodegradable, the LM-based biosensor will be integrated on the outer surface of the skeleton, and full health monitoring will be performed after the skeleton material has degraded. The LM bioelectrodes can be encapsulated with PEDOT:PSS, hydrogel or silver nanosheets.

### 4.2. Future Prospect of Integrated Vascular Stents Based on Liquid Metal

**Figure 6:**
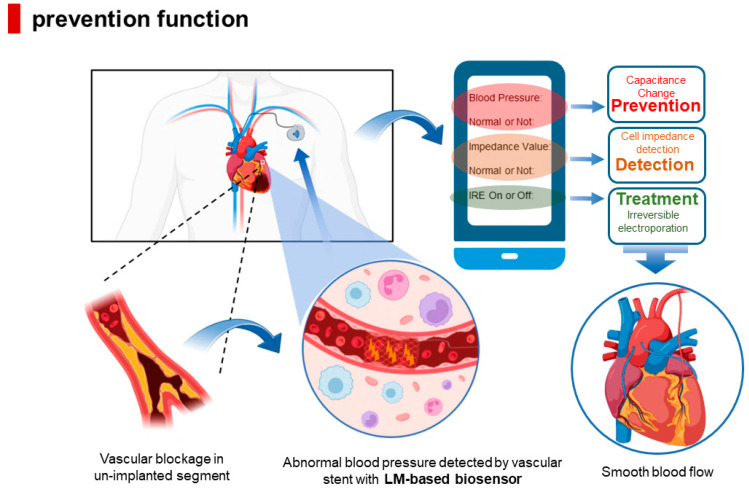
LM-based integrated vascular scaffold with prophylactic function.

The LM-based integrated vascular stent proposed in this paper has the function of blood pressure monitoring. As shown in Figure 6, the introduction of LM not only enables the combination of IRE technology with vascular stents, but also allows blood pressure measurement while inhibiting neointimal proliferation, thus achieving the triple effect of prediction, detection, and treatment.

#### 4.2.1. Detection of Neointimal Hyperplasia Thickness

With the introduction of LM bioelectrodes, the thickness of neointimal hyperplasia can be diagnosed by impedance detection of cells in real time. If patients do not follow the prescribed medication and improve their lifestyle habits (e.g., quit smoking, quit drinking and watch the diet), they may worsen the progression of atherosclerosis, which leads to in-stent restenosis. However, cardiovascular diseases are mostly emergencies, and if they are not treated in a timely manner they are very likely to be untreated. Therefore, if patients and physicians can monitor intimal hyperplasia in real time with impedance sensors formed by implanted electrodes, the mortality rate due to ISR can be greatly reduced.

#### 4.2.2. Permanent Prevention of ISR

The introduction of IRE technology may permanently prevent the development of late ISR. Compared to DES, both of which are permanent implants, vascular stents with IRE technology can still inhibit the proliferation of endothelial cells at a later stage. Thus, the integrated vascular stent not only effectively reduces the thickness of neointimal hyperplasia, but also solves the problem of “silent” late ISR. In addition, the long-lasting remote recording can provide surgical guidance to patients after the onset of other symptoms.

#### 4.2.3. Blood Pressure Monitoring

LM-based bioelectrodes are expected to enable the monitoring of blood pressure. Since the human body has blood vessels throughout the body, the diseased segments are not concentrated in only one place. If the implanted vascular stent enables blood pressure monitoring, then the blood pressure of the body will change when there is a blockage in a non-implanted segment. If this change is captured by the implanted integrated vascular stent, it can be fed back to the outside world in time for prediction. LM-based capacitive soft pressure microsensors have been shown to be useful for pressure measurement in microchannels.

#### 4.2.4. Reduce the Accumulation of Blood Cells

LM microelectrodes can be successfully integrated into microfluidic chips to capture and stretch red blood cells, which means that LM microelectrodes have the potential to drive blood cell movement and even induce blood cell death. If the bioelectrodes can control the degree of aggregation of blood cells on the surface of the vascular stent, then patients using this integrated vascular stent can greatly reduce or even eliminate the need for DAPT, which may be an excellent treatment option for patients at high risk of bleeding.

## 5. Conclusions and Outlook

Currently, vascular stents are optimized by loading drugs, modifying surfaces, and selecting biodegradable materials to reduce the rate of in-stent restenosis after implantation. However, none of these methods can prevent the emergence of advanced in-stent restenosis. In order to completely solve this problem, a new generation of vascular implant with intelligent sensing and real-time monitoring function is designed in this perspective. The new vascular stent can be composed of a drug-eluting stent or a biodegradable stent with an LM-based bioelectrode that combines high conductivity and stretchability. The LM-based flexible electrodes can be used for blood pressure monitoring (prediction), impedance detection (diagnosis), and electrically induced apoptosis (treatment). The electrodes also have the potential to control drug release, due to the fact that the LM-based electrodes can create an electric field within the blood vessels. The drug needs to be loaded onto the surface of degradable metal nanoparticles, e.g., LM nanoparticles, magnesium nanoparticles. The metal particles with the drug modified on the surface can be coated on a vascular stent, and the LM-based bioelectrode can be used to control the rate of drug release by adjusting the direction and magnitude of the electric field. In addition, the thickness of the electrode can be adjusted by using different preparation processes with the aim of obtaining an LM-based bioelectrode that is both soft and thin. The addition of LM-based electrodes provides a new direction for the development of next-generation vascular stents. There is an urgent need for further clinical trials regarding the new vascular scaffolds and in-depth exploration of their practical applications in the cardiovascular and even cerebrovascular fields.

## Figures and Tables

**Table 1:** The Company Names of All Relevant Brackets That Appear in This Perspective.

Name	Affiliated Company
Cypher stent	Cordis Corp–Johnson and Johnson company, New Brunswick, NJ, USA
Taxus stent	Boston Scientific, Natick, MA, USA
Taxus Liberte stent	Boston Scientific, Natick, MA, USA
Endeavor stent	Medtronic CardioVascular, Minneapolis, MN, USA
Xience V stent	Abbott Vascular, Markham, Ontario, Canada
COBRA PzF stent	Alta Biomaterials, Carlsbad, CA, USA
AMSorb stent	Beijing Advanced Medical Technologies, Ltd., Inc. Beijing, China
IBS stent	Lifetech Scientific, Shenzhen, GuangDong, China
Mirage BRMS stent	Manli Cardiology, Singapore

**Table 2:** Double-blind experiment results [20].

	Sirolimus Stent Set	BDS Set L	Paclitaxel Stent Set	BDS Set Z
4-year thrombosis rate	1.2%	0.6%	1.3%	0.9%
Number of thrombosis after 1 year	5	0	9	2

## Data Availability

Not applicable.
